# The characteristics of head and neck squamous cell cancer in young adults: A retrospective single-center study

**DOI:** 10.3389/pore.2023.1611123

**Published:** 2023-04-24

**Authors:** Mónika Révész, Ferenc Oberna, András Slezák, Örs Ferenczi, István Kenessey, Zoltán Takácsi-Nagy

**Affiliations:** ^1^ Multidisciplinary Head and Neck Cancer Center, National Institute of Oncology (NIO), Budapest, Hungary; ^2^ Center of Tumor Pathology Department, National Institute of Oncology (NIO), Budapest, Hungary; ^3^ Center of Radiotherapy, National Institute of Oncology (NIO), Budapest, Hungary; ^4^ Hungarian Cancer Registry, National Tumor Laboratory Project, National Institute of Oncology (NIO), Budapest, Hungary; ^5^ Department of Pathology, Forensic and Insurance Medicine, Semmelweis University, Budapest, Hungary; ^6^ National Tumor Laboratory Project, Budapest, Hungary; ^7^ Department of Oncology, Semmelweis University, Budapest, Hungary

**Keywords:** survival, risk factors, prognostic factors, head and neck cancer, young adults

## Abstract

We aimed to characterize clinical and prognostical factors of primary head and neck squamous cell carcinoma (HNSCC) in 85 young patients (≤39 years, median age: 37 years; between 2000–2018) in comparison with 140 institutional general HNSCC patients (median age: 61.5 years). The patient’s medical records were collected from the institutional database. The prevalence of smoking and alcohol consumption (65.8% and 48.1%) in the young group exceeded the regional population average but was below the institutional (86.4% and 55%) general HNSCC patient population. Primary tumor sites in the group of young patients were as follows: oral cavity (56.4%), oropharynx (17.6%), hypopharynx (11.7%), and larynx (14.1%). Cumulative five-year overall survival was 44.2% in the young group, but significantly better with early T (T1-2 vs. T3-4: 52.6% vs. 26.7%; *p* = 0.0058) and N0 status (N0 vs. N+: 65.2% vs. 32.3%; *p* = 0.0013). Young age, abstinence, earlier stage and laryngeal tumor site might predict a better prognosis. The age distribution and the high prevalence of traditional risk factors among the young patients as well as the predominance of oral cavity tumor localization suggest that the early onset of tumor development could be originated from the premature failure of the intrinsic protective mechanisms.

## Introduction

Head and neck squamous cell carcinoma (HNSCC) was estimated as the sixth most common cancer with approximately 800,000 new cases yearly worldwide based on the GLOBOCAN database (1). The annual incidence of 17.2/100,000 in Hungary is the highest in Europe (2). Although HNSCC usually occurs in patients over 60 years, in the past half century the incidence also increased in young adults. According to the literature, oral cancers constitute approximately 0.4%–3.6% of all cancers in patients under 40 years, and 6.7% under the age of 45 (3, 4). The cause of early-onset HNSCC is yet unclear (5). Certain authors indicated that traditional extrinsic risk factors (e.g., tobacco and alcohol consumption) may be similarly prevalent among older and younger individuals; however, the duration of the exposure in younger patients might not be sufficient to significantly increase the risk (5). The role of human papillomavirus (HPV) might also come into consideration (6, 7). Various factors might also play a role in the development of HNSCC in younger patients, such as genetic predisposition (e.g., Li-Fraumeni syndrome, Bloom’s syndrome, Fanconi anaemia, Wiskott-Aldrich syndrome, dyskeratosis congenita or further factors suggested by positive family history for HNSCC), an immunocompromised status, previous chemotherapy or irradiation, poor oral hygiene and dietary components, but their true relevance is yet unclear (6, 8). However, the increasing incidence might suggest the role of non-predetermined factors. Even the definition of young patient seems to be arbitrary, and varies between different reports (5, 9). Several studies aimed to compare the prognosis of young and older patients, but the results are conflicting (5, 9, 10). According to our knowledge, only few comprehensive reports have been published involving all sites of HNSCC (oral cavity, oropharynx, hypopharynx, larynx) in young patients. Our aim was to characterize the clinical features, the age distribution and the prevalence of classical risk factors in the young patient group in comparison with a non-preselected institutional general HNSCC population. In addition we would like to determine the impact of age and certain etiologic factors and tumor variables on 5-year survival rates and prognosis.

## Materials and methods

### Patients and study design

We summarized and analyzed the medical records of 85 young patients with histologically verified HNSCC between 2000 and 2018 based on the institutional medical database of *National Institute of Oncology, Budapest, Hungary*. Patients diagnosed under 40 years (≤39) of age were accepted as young, according to National Comprehensive Cancer Network (NCCN) Adolescent and Young Adult (AYA) Oncology’s guideline (11). We involved patients diagnosed and primarily treated in the *Multidisciplinary Head and Neck Cancer Center or Center of Radiotherapy*, as well as those who were admitted for adjuvant therapy (postoperative irradiation or chemoirradiation). According to the 10th revision of *International Classification of Diseases (ICD-10)* lip (C00), oral cavity (C01-C06), pharynx (C09; C10; C12; C13) and larynx (C32), with histologically verified squamous cell cancer morphology codes were included in our study (12). Patients with metastases of unknown primary tumors, as well as non-HNSCC (e.g., nasopharyngeal, salivary gland tumors, sinonasal carcinomas, thyroid tumors, lymphomas) tumors were excluded. Patient data (i.e., age, gender, drug and clinical history, tobacco and alcohol consumption, tumor site, stage, grade, and nodal status at the time of the diagnosis, time of death) were extracted from the hospital information system and the *National Cancer Registry*. Only those who had never smoked were considered non-smokers. Drinkers were defined as alcohol consumption at or above the *World Health Organization (WHO)* medium risk category (>40 g alcohol for men, or >20 g for women), which constitutes approximately a bottle of beer or >2–3 dL of wine on a daily basis (13). Each tumor was staged according to the 7th edition of TNM criteria of the Union for International Cancer Control (UICC) (14) and WHO classification was used to assess histological type and grading (12). Paraffin-embedded tissue sections were also used for the confirmation of the diagnosis and immunohistochemical staining for p16^INK4a^ antigen with Roche CINtec® p16 (E6H4^TM^) antibody for detection of this antigen (used according to the manufacturer’s recommendation, Roche, Basle, Switzerland) in the *Center of Tumor Pathology Department of National Institute of Oncology*. The clinicopathological data of our study population were compared with an institutional group of 140 consecutive general HNSCC patients from year 2014.

The study was conducted under the ethical permission of the Scientific and Research Ethics Committee of the Medical Research Council (approval number: BMEÜ/3719- 1 2022/EKU) in accordance with the Helsinki Declaration of 1975, as revised in 2008.

### Follow-up

Recurrence was defined as the local or regional return of cancer after the patient was declared disease-free. Residual tumor was defined as histologically positive resection margins after surgical therapy, or tumor suspect soft tissue detected by radiological imaging (Computed tomography/CT/, Magnetic resonance imaging/MRI/, Positron emission tomography/PET/-CT) within 3 months from the end of the radio(chemo)therapy or other conservative treatment (in certain cases the residual tumor was also proved by histology). We also diagnosed locoregional residual disease if the locoregional recurrence occurred within 3 months after the first surgical treatment regardless of the surgical margin status. Overall survival (OS) was determined as months between the date of initial diagnosis and the last follow-up or death.

### Statistical analysis

Chi-square test (CS) was used to compare the composition of groups. Fisher’s exact test (FE) with Bonferroni correction of the alpha values was used as a post-hoc test for multiple comparisons. Kolmogorov-Smirnov test was used to assess the normality of distributions. Student’s t-test or Mann-Whitney U-test (MWU-test) was used to compare independent variables with normal or non-normal distribution, respectively. Survival curves were depicted according to the Kaplan-Meier method (KM), significance was analyzed by log-rank statistics (LR). Univariate and multivariate analyses of prognostic factors were done using the Cox’s regression model. A *p*-value < 0.05 was accepted as statistically significant. KyPlot 5.0 (KyensLab Inc., Tokyo, Japan) and Statistica 14.0.1.25 (TIBCO Software Inc., Palo Alto, CO) were used for statistical analysis.

## Results

### Tumor characteristics of young patients

Among young patients male predominance (78.9%, male/female: 67/18) was found. The median age at the time of the diagnosis was 37 years (range 21–39) and two-third of the patients were 35–39 years old. For comparison we involved an institutional general HNSCC group (male/female: 104/36; median age: 61.5 years, range 34–88). The most common primary tumor locations were the lip and the oral cavity in contrast to the general patient population (48/85 [56.47%] vs. 27/140 [19.28%]; *p* < 0.001, CS). The most common subsite was the tongue in contrast to the oral cavity tumors of the general HNSCC patients (26/48, [54.1%] vs. 6/27, [22.2%], *p* < 0.007, CS). In the oral cavity subgroup 6 of 48 young patients (12.5%) and 3 of 27 general HNSCC patients (11.1%) presented with lip cancer, the difference was none-significant (*p* = 0.85, Chi-square test). Fifty-seven of 85 young patients had early (T1-T2) primary tumor status at the time of the diagnosis, which is significantly different from the control group (57/85 [67%] vs. 66/140 [47.1%], respectively, *p* < 0.037). Fifty-four (63.5%) had cervical lymph node involvement (N+) according to the physical examination and radiological imaging (ultrasound, CT scan, MRI). Lymph nodes were seldom involved in laryngeal cancer (N0: 83.3%, 10 of 12). None of the patients had distant metastasis (M) at the time of the diagnosis. Well-differentiated tumors are significantly more common among young patients than in the control group (55/73 [75.3%] vs. 73/119 [61.3%], respectively, *p* < 0.05). Detailed characteristics of all study groups are reported in Table 1.

**TABLE 1:** Clinical characteristics and treatment methods of the young patients and the institutional control group.

	Young patients (*n* = 85)	Control group (*n* = 140)	*p* (Chi-square)
Sex			0.44
Male	67 (78.8%)	104 (74.3%)	
Female	18 (21.2%)	36 (25.7%)	
Median age (yr)	37	61.5	**0.001**
Interquartile range (yr)	33–39	56.75–67	
Smoking history			9.3*10 ^−^ **^5^**
pos	52 (65.8%)	123 (87.9%)	
neg	27 (34.1%)	17 (12.1%)	
NA	6	0	
Alcohol consumption			0.235
pos	38 (48.1%)	79 (56.4%)	
neg	41 (51.8%)	61 (43.6%)	
NA	6	0	
Localization			2.1*10 ^**−**^ **^7^**
Lip, oral cavity	48 (56.5%)	27 (19.3%)	
Mesopharynx	15 (17.6%)	35 (25%)	
Hypopharynx	10 (11.8%)	34 (24.3%)	
Larynx	12 (14.1%)	44 (31.4%)	
Primary tumor size			**0.037**
T1	24 (28.2%)	28 (20%)	
T2	33 (38.8%)	38 (27.1%)	
T3	12 (14.1%)	30 (21.4%)	
T4	16 (18.8%)	44 (31.4%)	
Nodal status			0.83
N0	31 (36.5%)	49 (35%)	
N1	16 (18.8%)	24 (17.1%)	
N2	33 (38.8%)	54 (38.6%)	
N3	5 (5.9%)	13 (9.3%)	
Stage			0.375
I	16 (18.8%)	16 (11.4%)	
II	8 (9.4%)	10 (7.1%)	
III	18 (21.2%)	31 (22.1%)	
IV	43 (50.5%)	83 (59.3%)	
Grade			**0.043**
1–2	55 (75.3%)	73 (61.3%)	
3–4	18 (24.7%)	46 (38.6%)	
NA	12	21	
Perineural spread			0.37
Pos	13 (38.2%)	17 (29.3%)	
Neg	21 (61.8%)	41 (70.7%)	
NA	51	82	
Vascular invasion			0.23
Pos	10 (28.6%)	24 (40.7%)	
Neg	25 (71.4%)	35 (59.3%)	
NA	50	81	
Primary treatment			0.11
Surgery	15 (17.6%)	12 (8.6%)	
non-surgery	28 (32.9%)	46 (32.8%)	
Combined	42 (49.4%)	82 (58.5%)	

NA: not available.

The significant values were marked with bold characters.

### Prevalence of potential risk factors in young patients

Data on smoking and alcohol consumption could be found in the medical records of 79 young patients. Fifty-two (65.8%) of them were (current or previous) smokers, 38 (48.1%) consumed alcohol and 33 (41.7%) enjoyed both risk factors. However, smoking was significantly less prevalent in the young group than in the general HNSCC patient population (65.8% vs. 86.4% respectively, *p* < 0.001, CS). In contrast, the alcohol consumption was similar in the young and the general HNSCC group (48.1% vs. 55.0%, respectively, *p* < 0.0001 CS) (Table 1). The p16 status was tested in 65 young and 36 control patients. The rate of positivity was 20% (*n* = 13) and 11.1% (*n* = 4) respectively, no significant difference was shown between the two groups (*p* = 0.25, Chi-square test).

### Front-line therapy and histological parameters

Fifty-seven of the 85 patients (67%) underwent surgical therapy, 85.4% of those with lip and oral cavity cancer, 75% with laryngeal cancer, 40% with hypopharynx, 20% with mesopharynx cancer. Forty (70.2%) of the 57 patients underwent R0 resection, among them 10 patients with close R0 resection (the free margin is < 5 mm). In 29.8% (*n* = 17) of patients R1 resection was performed.

Two patients (20%) with close R0 resection, and 6 patients (35.2%) with R1 resection underwent a follow-up operation. The indications for postoperative irradiation were pT3 or pT4 stage, perineural spreading, vascular invasion, extranodal extension and close or positive surgical resection margins. Altogether 42 patients (73.6%; *n* = 31 patients with R0; *n* = 11 with R1 resection) received adjuvant therapy after the surgical therapy (Table 1.).

### Overall survival (OS) of young patients

**FIGURE 1:**
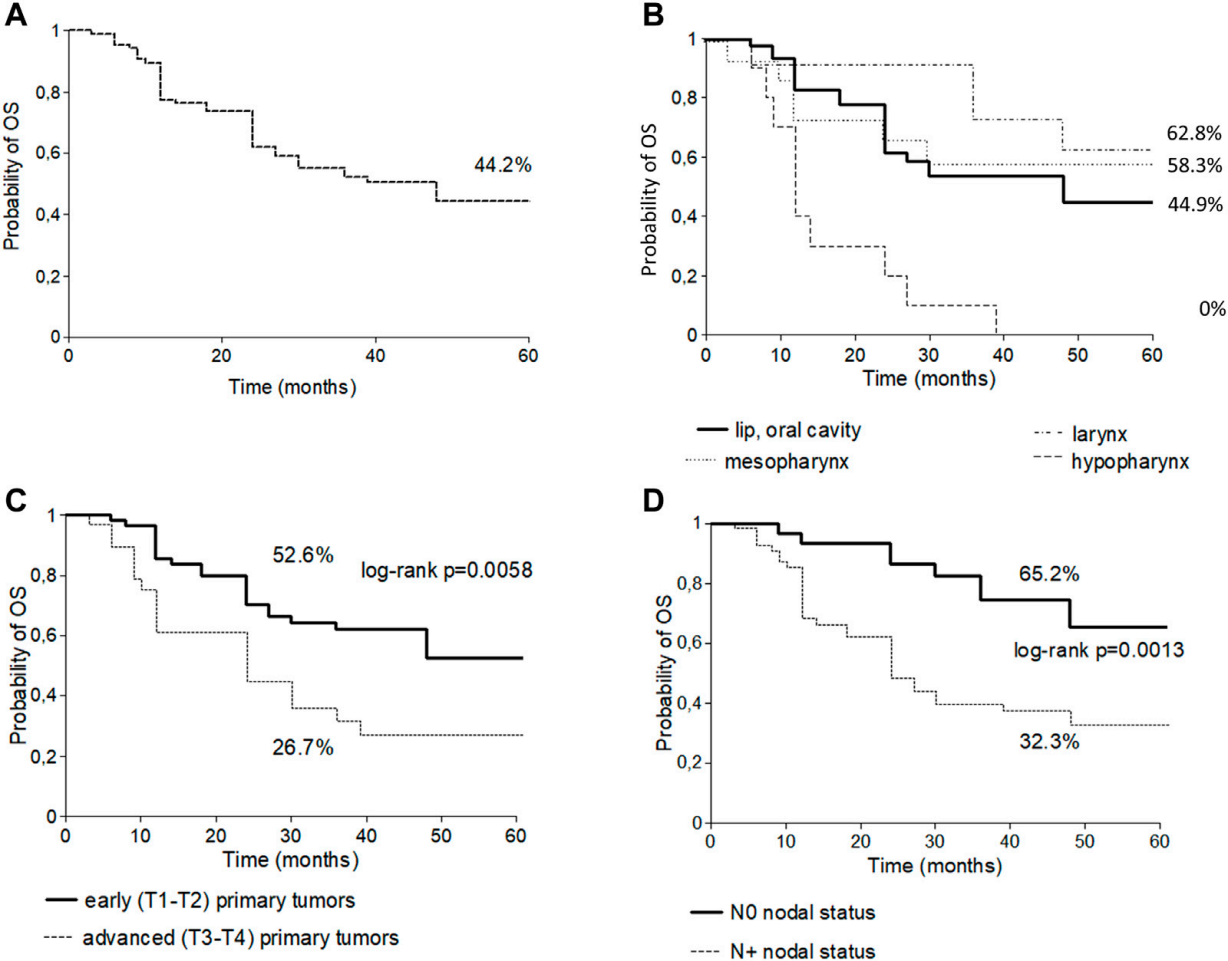
Five-year overall survival curve of all sites of head and neck cancers in young patients **(A)**; Five-year overall survival curve of subtypes of head and neck cancers in young patients **(B)**; Five-year overall survival curve stratified by T stage in young patients **(C)**; Five-year overall survival curve stratified by N stage in young patients **(D)**.

The median follow-up time since the diagnosis of cancer was 28.5 months (range 3–228). Cumulative five-year OS for all head and neck sites was 44.2% (Figure 1A), 44.9% for lip and oral cavity (*n* = 47), 58.3% for oropharynx (*n* = 15), 0% for hypopharynx (*n* = 10) and 62.8% for larynx (*n* = 12) carcinoma. Only the subgroup with hypopharynx carcinoma differed significantly from all others (Figure 1B).

The 5-year OS was analyzed according to risk factors. Significantly better OS was observed in the alcohol abstinent group (59.6% vs. 32.7%; *p* = 0.0297). The 5-year OS was significantly better in patients with early, than in advanced T status (T1-2: 52.6% vs. T3-4: 26.7%; *p* = 0.0058) (Figure 1C) and in patients with N0 vs. N+ nodal status (65.2% vs. 32.3% respectively; *p* = 0.0013) (Figure 1D).

The cumulative 5-year survival for all tumor sites was significantly better in the young than in the control group (44.2% vs. 32% respectively, *p* = 0.005).

Univariate Cox regression model for the entire study population revealed that young age, a non-smoker or abstinent status, as well as laryngeal tumor site might predict a significantly better prognosis. Data adjustment for multivariate Cox regression model proved that from the aforementioned parameters young age, abstinence and earlier stage remained independent prognostic parameters, which were associated with more favorable outcome (Table 2). Contrarily, alcohol consumption, older age and advanced stage almost doubled the relative risk. When analyzing the young group separately, an alcohol-abstinent status, as well as advanced stage proved significant predictors of the prognosis. All the significancies and trends in the young age group were in line with those in the entire study group (Table 3).

**TABLE 2:** Univariate and multivariate Cox regression model for the assessment of prognostic factors in the entire (young patients and control group) study population (RR = relative risk).

	Univariate model		Multivariate model	
	RR (95% CI)	*p*	RR (95% CI)	*p*
Young group (vs. control)	0.497 (0.309–0.797)	0.004	0.617 (0.426–0.894)	0.011
Male sex (vs. female)	0.837 (0.525–1.334)	0.454	0.708 (0.474–1.057)	0.091
Smokers (vs. non-smokers)	1.799 (1.037–3.121)	0.037		
Alcohol (vs. abstinent)	1.641 (1.073–2.508)	0.022	2.007 (1.394–2.888)	1.8*10^−4^
Localization (vs. oral cavity)				
mesopharynx	0.877 (0.448–1.714)	0.308		
hypopharynx	0.814 (0.456–1.453)	0.164		
Larynx	0.398 (0.221–0.718)	0.005		
Grade 3/4 (vs. 1/2)	0.854 (0.581–1.255)	0.421		
Stage IV (vs. I-III)	2.454 (1.605–3.752)	3.4*10^−5^	1.883 (1.343–2.64)	2.4*10^−4^

**TABLE 3:** Univariate and multivariate Cox regression model for the assessment of prognostic factors among young head and neck squamous cancer patients.

	Univariate model		Multivariate model	
	RR (95% CI)	*p*	RR (95% CI)	*p*
Male sex (vs. female)	1.146 (0.531–2.472)	0.728	0.592 (0.208–1.688)	0.327
Smokers (vs. non-smokers)	1.796 (0.85–3.797)	0.125	1.746 (0.664–4.589)	0.258
Alcohol (vs. abstinent)	1.973 (1.029–3.783)	0.041	1.433 (0.61–3.369)	0.409
Tumor site (vs. oral cavity)				
mesopharynx	1.683 (0.464–6.113)	0.282	0.434 (0.05–3.79)	0.043
hypopharynx	4.08 (1.898–8.769)	3.1*10^−5^	3.602 (1.404–9.244)	3.9*10^−5^
Larynx	0.546 (0.188–1.582)	0.063	0.325 (0.071–1.491)	0.189
Grade 3/4 (vs. 1/2)	0.734 (0.333–1.618)	0.444	0.939 (0.396–2.227)	0.887
Stage IV (vs. I-III)	1.904 (1.026–3.535)	0.041	1.948 (0.908–4.179)	0.087

### Locoregional control

Residual disease was reported in 42.3% (*n* = 36) of the 85 patients. The histological analysis of the surgical materials showed positive margins in 17 cases, and residual disease was also detected in 15 cases after the completion of other treatment modalities (e.g., definitive radio(chemo)therapy, induction chemotherapy+ radio(chemo)therapy) by radiological imaging. We also considered four further cases of locoregional recurrences to constitute residual disease, as recurrence occured within 3 months (average of 10 weeks, range: 8–12) after surgical treatment with tumor-free margin (R0) status.

Recurrent disease was observed in 24.7% (*n* = 21) of the 85 patients (12 local, 4 regional, 3 distant metastasis and 2 locoregional recurrence) after that the patient was defined as disease free, and occurred within an average of 24 months after the diagnosis (local: 15.14 months/range 4–48/; regional: 9 months/range 4–20/; distant: 20 months/range 14–24/). The incidence of second primary cancers (lung, oropharynx and tongue) was 4.7% (*n* = 4).

## Discussion

Previous studies confirmed that smoking, alcohol consumption and HPV are the most important risk factors of HNSCC (5, 6). However, these carcinogens are known for their delayed effect. As the time of exposure is limited in young patients, additional factors are suspected in the background. This fact suggested that, compared with the general population with HNSCC, the young group might show a different pattern. The importance of our examination is that the majority of HNSCC studies did not handle young patients separately. The proportions of smokers and alcohol consumers, as well as the gender distribution and the proportions of certain primary tumor locations in our control HNSCC patient group were practically identical with large-scale international hypothesis-free epidemiological HNSCC studies (15, 16). We found that, in contrast to the general HNSCC populations, the oral cavity proved to be the most common tumor site in young patients. In addition, the majority of patients were diagnosed in an early tumor stage. In parallel with previous works, among our examined patients an advanced T and N status were associated with significantly worse prognosis, which underlines the relevance of early recognition and treatment (15). This also corroborates with our findings that young age was associated to relatively better outcome. The location-based 5-year survival analysis showed significantly worse survival in the hypopharynx subgroup than in the other subgroups in young patients. However, the comparability of tumor site specific survival data was limited due to the small number of the subgroups. The low prevalence of p16 positivity suggested that HPV infection is unlikely to play a relevant role in our patient groups.

There is also an unanswered question of whether young HNSCC patients form a distinct disease entity (17). A recent report by Pickering et al. suggested, that oral tongue SCCs from younger and older patients are genomically similar (18). This might indicate, that the younger and older patients might rather differ in their susceptibility to carcinogenesis than the characteristics of the tumors. Intrinsic (mainly genetic) and environmental factors might act synergically. Tobacco use and alcohol consumption are well-known causative factors. Although the duration of the exposure is necessarily shorter (by decades) among young patients, prevalence of tobacco and alcohol consumption in our study population was significantly higher compared to the >15 years old regional general population (30.6% (19); 32.3% (20) respectively. A recent Hungarian report of oral squamous cell cancer cases has also stated that the number of smokers was high in both the older and younger oral cancer patient groups (21) however, the definition of drinkers is often used inconsistently, which makes the comparisons less exact between various studies. We hypothesize that the sufficiency of lower cumulative doses of these harmful agents suggests an increased vulnerability of this population to lifestyle-related risks, which might originate from a diminished damage control on the cellular level, or a weaker capacity to eliminate tumor cells by the innate immune responses. This concept is suggested by Wang et al. who linked reduced DNA repair capacity with tobacco -related carcinogenesis and also Kostrzewska-Poczekaj et al. who identified polimorphisms of DNA repair genes in young HNSCC patients (22, 23). Our hypothesis might be in line with previous reports regarding other malignancies associated with extrinsic factors: candidate genes involved in the regulation of cellular proliferation and the prevention of DNA damage have already been identified in association with early onset lung cancer (24, 25), and a distinct immune signature of early and late onset colorectal cancer has also been described (26). On the other hand, the higher proportions of non-smoking and abstinent patients among young patients are unexplained by the traditional carcinogenic theories. Thus, the young HNSCC population might not be homogenous (10). For instance, Lee et al. suggested that young patients with HNSCCs linked primarily to extrinsic risks (smoking and alcohol) might carry a more favorable prognosis than less heavily exposed individuals (27). However, the latter group seems to remain a minority in our study population. Additionally, in Hungary all the three major risk factors are endemic, which does not allow to determine strict risk groups (28). The age distribution of our patients is heavily distorted towards the cut-off value of 39 years, which might indicate that the majority of them might represent an extreme value of the general patient population. Of note, five of the sixteen young patients (31.2%) who lacked any traditional cancer risk factors (tobacco and/or alcohol use and also p16 positivity) were immunocompromised (three after bone marrow transplantation, one with Crohn’s disease, one during pregnancy) however their number was insufficient for a subgroup analysis. Previous studies also found that chronic immunodeficiency (such as immunosuppression following organ transplantation or HIV) is associated with a higher incidence of oral squamous cancer in young patients; however, the available results are contradictory (4). Additionally, the metaanalysis of van Monsjou et al. identified different hereditary syndromes, such as Fanconi anemia and Bloom’s syndrome as causative factors in a subgroup of young-onset HNSCC, but our examinatory sample was not able to reflect to this question (6).

In summary, in our study we found that young patients with HNSCC might represent an extreme value within the spectrum of the general HNSCC patient population. This is suggested by their age distribution and the high prevalence, but necessarily lower cumulative doses of traditional risks. Thus HNSCC in young adults is unlikely to be based on endogenous risks directly for carcinogenesis (i.e., tumor predisposition syndromes) but they might rather show an exceeded susceptibility to classical risk factors.

Although the etiological factors might be similar, young patients do have a better prognosis according to our results. Given that the most common tumor site was the oral cavity in young patients we should highlight the importance and feasibility of screening.

## Data Availability

The raw data supporting the conclusion of this article will be made available by the authors, without undue reservation.
